# The delayed management of main arterial injuries in extremity trauma: Surgical challenges and outcomes

**DOI:** 10.12669/pjms.291.2619

**Published:** 2013

**Authors:** Liguo Shi

**Affiliations:** Liguo Shi, MD, Department of Surgery Emergency, Tianjin 4^th^ Centre Hospital, Tianjin, 300140, China.

**Keywords:** Arterial Injuries; Ischaemia time; Amputation rate

## Abstract

***Objective: ***To analyse the early outcome of main arterial injuries with delayed treatment in extremity trauma and help vascular surgeons in determining proper treatment strategy for such injuries.

***Methodology:*** Forty-three patients with delayed treatment of main arterial injuries during May 2003 and January 2008 were reviewed retrospectively.

***Results: ***In 43 patients, injuries were caused by blunt trauma in 26 cases and penetrating trauma in 17 cases. The maximum ischaemia time was 38 hours and the minimum was 13 hours. Eight patients underwent primary amputations and four patients underwent secondary amputation. There was no perioperative mortality, while wound infection occurred in five patients, followed by graft occlusion in four patients, arteriovenous fistulae in two patients and pseudoaneurysm in one patient.

***Conclusion:*** The delayed intervention in main arterial injuries is associated with higher risk of amputation, while the suitable surgical techniques may decrease the risk of limb loss. Viable limbs should be revascularized in otherwise stable patients even with long periods of ischaemia.

## Introduction

 Main arterial injuries are uncommon but potentially fatal lesions in extremities trauma. Early diagnosis and immediate intervention are mandatory to save the extremities and lives of the patients.^1^ Many factors, such as extent of soft tissue damage, the capacity of collaterals and pre-existing arterial disease may affect the clinical outcomes, while the ischaemia time is more critical, and more than eight hours of ischaemia time are associated with increased rates of limb loss.^2^ Therefore, the rapid detection, localization and characterisation of arterial injuries are essential for the effective management and treatment.

 However, limited facilities to manage vascular injuries in some small hospitals, coupled with delays in diagnosis and transfer to large hospitals, pose major challenges with regards to optimum management of these injuries.^3^ Such limitations may be seen in many parts of the world, and the analysis and summary of the patients with delayed management may be helpful for vascular surgeons in determining proper treatment strategy for such injuries.

 In the present study, we have evaluated our experience with a subset of patients with delayed late intervention of main arterial injuries in extremities.

## Methodology

 One thousand and thirty-nine patients of arterial injury presented in our hospital between May 2003 and January 2008. Among 1039 patients, 43 patients with delayed intervention were reviewed retrospectively. The data including etiology of injury, location and severity of vascular and orthopaedic injuries, methods of vascular repair, amputation rates and existing complications were collected. 

**Table-I:** Distribution of vascular injuries.

*Localization*	*Artery*	*Artery and vein*	*Total*
Axillary	2	1	3
Brachial	6	5	11
Femoral	5	3	8
Popliteal	7	4	11
Anterior tibial	3	2	5
Posterior tibial	2	3	5
Total	25	18	43

 Our study population consisted of patients in which the vascular injury occurred in extremities, and the period between revascularization and vascular injury exceeded eight hours. Patients were identiﬁed using the International Classiﬁcation of Diseases (ICD) for injuries and procedures. This study was approved by the Ethics Committee of the Tianjin 4TH Central Hospital.

 The diagnosis of arterial injury was made according to physical exam, with the assistance of hand Doppler, computed tomography angiography or surgical exploration. Primary amputation was performed in patients with non viable limbs. Artery injuries were managed mainly by end-to-end anastomosis or interposition vein grafting, using autologous reversed long saphenous vein from the contra- lateral limb. The repair of vein was made in the case of the axillary, femoral and popliteal veins using direct repair or vein graft techniques, and other venous injuries were ligated. 

 Arterial repair preceded orthopaedic repair, and orthopaedic injuries were managed with open reduction and internal fixation, or external fixation devices. In terms of nerve injuries, primary repair was performed if the wound was not contaminated, or the delayed repair was performed at 2-3 weeks.

 Postoperatively the patients were maintained on intravenous prophylactic antibiotics and venous thromboprophylaxis with low molecular weight heparins.

**Table-II:** Applied surgical interventions for artery repair.

*Surgical intervention*	*Number*	*Ratio (%)*
Saphenous vein graft interposition	11	25.6
End-to-end anostomosis	20	46.5
Lateral arterioraphy	7	16.3
PTFE graft interposition	3	6.9
Ligation	2	4.7

## Results

 There were 43 patients in the study. Thirty-two were males and 11 were females. The average age was 42.2 years (range from 32.3 to 54.5 years), and the mean follow-up is 11 months (range from two months to 17 months). The ischaemia time was defined as the period from the approximate time of injury to the time at which the patency of the injured vessel was restored at surgery. In this series, the maximum duration was 38 hours and the minimum was 13 hours. 

 The etiology of vascular injury was blunt trauma in 26 cases and penetrating trauma in 17 cases. Blunt trauma was caused by road traffic accidents in 21 cases and falls in five cases, and penetrating trauma was caused by industrial injuries in 11 cases and stabbings in six cases. The distribution of vascular injuries and the applied surgical interventions can be seen in Table-I, Table-II and Table-III. The concomitant orthopaedic injuries occurred in 19 cases, the distribution and treatment methods of which can be seen in Table-IV. The concomitant peripheral nerve injury occurred in 22 cases, which were repaired primarily. The distribution and treatment methods of nerve injury can be seen in Table-V. In addition, fasciotomy was performed simultaneously in surgery of vessel repair in 19 cases.

**Table-III:** Applied surgical interventions for venous repair.

*Surgical intervention*	*Number*	*Ratio (%)*
Saphenous vein graft interposition	5	27.8
End-to-end anostomosis	9	50
Ligation	4	22.2

**Table-IV:** Skeletal injuries and treatment methods.

*Localisation*	*Number*	*External fixation*	*Internal fixation*
Femur fracture	3	-	3
Tibia fracture	7	-	7
Humerus fracture	3	-	3
Shoulder dislocation	1	1	-
Elbow dislocation	2	1	-
Hip dislocation	2	2	-
Knee dislocation	1	1	-

**Table-V:** The distribution and treatment methods of nerve injury.

*Localisation*	*Number*	*Direct nerve suture*	*Autologous nerve graft*
Median nerve	2	2	-
Ulnar nerve	3	3	-
Radial nerve	2	1	1
Femur nerve	5	4	1
Tibial nerve	6	4	2
Commonperoneal nerve	4	4	-

 No patients within our group died postoperatively. Eight patients presenting with ischaemia vascular injuries were found to have nonviable limbs and were offered primary amputation. Four secondary amputations were performed due to graft failure. The amputation rate in this series is 27.9%. While, among the 996 patients treated within 8 hours after injury, amputation were performed in 61 patients and the amputation rate is 6.1%, which is significantly lower than that in this series (p<0.05). In addition, infections, deep vein thrombosis, arteriovenous fistulae and pseudoaneurysm were also noted. Wound infection was the common complications that occurred in five patients, followed by graft occlusion in four patients, arteriovenous fistulae in two patients and pseudoaneurysm in one patient. Wound infection was treated by daily dressings and change of antibiotic as per swab culture sensitivity. Graft occlusion, arteriovenous fistulae and pseudoaneurysm were treated by surgeries.

## Discussion

 In the present study, we reviewed retrospectively the 43 cases with delayed management of main arterial injuries in our hospital, and the aggregate experience may provide a more defined constellation of the etiologies, optimal management strategies, and outcomes of such an injury.

 In vascular surgery mortality and morbidity of the vascular injury is highly related to the duration between the injury and surgical intervention.^4^ More than eight hours of ischaemia time were associated with increased risk of limb loss, and the amputation rates climb as ischaemia times lengthen.^2^ In the present study, the amputation rate in patients with delayed intervention is significantly higher when compared to those treated within 8 hours after injury, demonstrating the increased risk of limb loss accompanied with delayed management. Subsequently, the optimal revascularization time should be in eight hours after injury. However, the optimal opportunities were missed in many cases because of delays in diagnosis or transfer to large hospitals. In the present study, eighteen patients were delayed because of transferring from peripheral hospitals, among which seven patients were performed amputation. 

 Another reason for delay was missed diagnosis in local hospitals, especially when concomitant fracture or dislocation occurs. In the present study, of 25 cases with missed diagnosis 13 had bone injuries, and the swollen limbs resulted from fracture or dislocation were apt to the missed diagnosis. Fractures and dislocations were not uncommonly complicated by arterial injuries, most of which were diagnosed and treated without delay. However, arterial injuries associated with orthopaedic trauma were not always recognized. Amputation rates as low as 4% have been reported with isolated arterial injuries, while rates as high as 61% have been seen when combined vascular, skeletal, and soft tissue injuries are present.^5^

 Thus, the emergency physician in the first place must have a high level of suspicion. Patients with any hard sign of arterial injury, i.e., pulsatile bleeding, absent distal pulse, limb ischaemia, expanding haematoma, shock with ongoing bleeding, bruit or thrill over area of injury should be taken directly to the operating room without further diagnostic testing^1^, and the patients without hard signs, unless easily palpable pulses are present distal to the bony injury, arterial trauma must be assumed to have occurred, we would suggest thorough physical exam, with the assistance of hand Doppler, computed tomography angiography or surgical exploration, be performed to rule out the vascular injury.

 In surgery, the timing of orthopaedic fixation in concomitant bone injury is controversial; some surgeons advocated that skeletal fixation should be performed prior to vessel repair6, while others held the opposite views.^7^^,^^8^ We support the viewpoints that vascular repair should be carried out before bone stabilisation in order to decrease duration of ischaemia^8^, while vascular repairs should be inspected following vigorous repair of fractures or dislocations to ensure vessels remain patent.^2^ Moreover, we usually repair the concomitant vein injury in proximal limb, such as axillary vein, brachial vein and the popliteal vein, which is helpful in keeping the repaired artery open and preventing postoperative edema.^5^

 Many authors have highlighted the importance of fasciotomy in the vascular reconstruction^9^^-^^11^ because the soft tissue trauma, crush injury and venous injury result in the higher risk of compartment syndrome^8^^,^^9^, especially when the time of reconstruction exceeds the four to six hours post injury.^12^ As a result, some scholars suggest that fasciotomy performed at the time of arterial repair, but before the development of compartment syndrome, may lower amputation rates especially in patients with long preoperative delays.^2^ In the present study, fasciotomy was performed in 19 cases simultaneously in vascular repair. The present study was not a large sample, controlled one, but we suggest the fasciotomy would play an important role in preventing complications of vascular injury.

Moreover, the popliteal artery injury occurred in 11 patients in the present series, among which primary amputation were performed in four patients, and secondary amputation in one patients, demonstrating the high amputation rate in popliteal artery injuries, and this may be attributed to the prolonged ischaemia time. In addition, we found the similar vascular injuries may have different outcomes, even in patients with popliteal artery injury. In the present series, we had four patients of popliteal artery injury with the similar locations, soft tissue injury and ischaemia time. Two of them got successful vessel repair, while the others were performed primary amputations unfortunately, because of nonviable limbs. Although the ischaemia time is critical, other factors, such as extent of soft tissue damage, the capacity of collaterals, pre-existing arterial disease may affect the clinical outcomes of vascular injuries. We attributed the different clinical results to the collaterals of popliteal artery and the paucity of collaterals around the knee may result in the early necrosis of limbs.^11^

 Some scholars^4^ suggest the survival of the limb may be improved by the presence of collaterals, and in the study on 102 patients of popliteal artery injury, Melton^13^ found the overall amputation rates were significantly higher in patients with ischemic extremities compared to those with some evidence of collateral flow. Wagner et al^14^ found a lack of correlation between ischaemia time and outcome in vascular injury. The presence of collaterals alone may not be able to support the function of the limb in cases of arterial injury to major limb vessels, but they may decrease the risk of limb loss. Additionally, the time since injury, may not necessarily reflect the actual period of ischaemia because of the presence of collateral circulation.^3^ Subsequently, we suggest all viable limbs with continued ischaemia be revascularized in otherwise stable patients even with long periods of ischaemia.

 In conclusion, the delayed intervention in arterial injuries is associated with higher risk of amputation. While, the suitable surgical techniques, such as liberal application of fasciotomy, suitable cure of other accompanying pathologies as well as vascular repair prior to orthopaedic repair may decrease the risk of limb loss. In addition, all viable limbs should be revascularized in otherwise stable patients even with long periods of ischaemia.
